# In-Fiber Optic Salinity Sensing: A Potential Application for Offshore Concrete Structure Protection

**DOI:** 10.3390/s17050962

**Published:** 2017-05-04

**Authors:** Dong Luo, Peng Li, Yanchao Yue, Jianxun Ma, Hangzhou Yang

**Affiliations:** 1 School of Human Settlements and Civil Engineering, Xi’an JiaoTong University, Xi’an 710048, China; jerrylee@xjtu.edu.cn (P.L.); yuey@xjtu.edu.cn (Y.Y.); majx@xjtu.edu.cn (J.M.); 2 Photonics Research Centre, University of Malaya, Kuala Lumpur 50603, Malaysia; yanghz2005@hotmail.com

**Keywords:** concrete structure, salinity, fiber optic sensor

## Abstract

The protection of concrete structures against corrosion in marine environments has always been a challenge due to the presence of a saline solution—A natural corrosive agent to the concrete paste and steel reinforcements. The concentration of salt is a key parameter influencing the rate of corrosion. In this paper, we propose an optical fiber-based salinity sensor based on bundled multimode plastic optical fiber (POF) as a sensor probe and a concave mirror as a reflector in conjunction with an intensity modulation technique. A refractive index (RI) sensing approach is analytically investigated and the findings are in agreement with the experimental results. A maximum sensitivity of 14,847.486/RIU can be achieved at RI = 1.3525. The proposed technique is suitable for in situ measurement and monitoring of salinity in liquid.

## 1. Introduction

Salinity is a significant property of natural water and industrial wastewaters. Salinity detection and measurement has widespread applications and a great realistic significance in the protection of the marine environment, oceanic, mariculture, water source quality, and concrete structures [1]. In concrete structures, Portland cement plays a key function in maintaining the health of the structure, however, it is an alkaline material. Ideally, it is the preferred choice to place the concrete structure at, or near, an alkaline environment to preserve and maintain the integrity of the concrete structure; however, it is challenging in the scenario where concrete structures are built in a marine environment. Due to the pores and micro-cracks in the concrete, the saline solution can infiltrate into the concrete structures. The infiltrated saline solution is a mild acid that lowers the pH of the concrete and compromises the adhesive ability of the cement that holds the concrete structures together. Furthermore, the saline solution also attacks the concrete paste and increases the pore size, which allows more infiltration of the saline solution and other chemical substances into the deeper parts of the concrete structures, thus exacerbating the freeze/thaw cycle damage. When the salt reaches the steel reinforcement, the thin protective layer of iron oxide surrounding the reinforcement has limited ability against the corrosive attack. Eventually, the protective layer will fail and the corrosion will aggravate. Generally, the created corrosion products absorb water and their volume can be 3–6 times larger than their original size; the resulting pressure from the growing corrosion can cause the surrounding concrete to crack and break [2]. Considering the connection between the solution salinity, the pH of the solution, and the strength of the concrete paste, the salinity concentration can serve as a good indicator for diagnosis and structural monitoring of the concrete under corrosion.

Electrical conductivity is a commonly used parameter for liquid salinity measurement, however, its relationship to the mass of dissolved salts are subject to several other variables and uncertainties, such as the ionic substances in the solution may have different conductivity from the sodium chloride salt solution, the amount of undissolved salts in a saturated saline solution, and the local temperature of the solution where the measurement is made. The operation of the electrical conductivity meter for measurement of the salinity requires ensuring the calibration solution is fresh. If the conductivity meter measures a wide range of conductivities, the calibration solution should have a similar conductivity range [3]. 

Abbe refractometers are among the most commonly used system for refractive index (RI) measurement, which is based on determination of the critical angle of an analyte on an optical prism. Generally, small test samples are required and consumed for every measurement and it may not be suitable for an application where in situ measurement and monitoring are required.

Owing to the inexpensive and efficient delivery of a signal, optical fiber devices are commonly used in industrial sensing applications [4,5,6,7,8]. The fiber optic sensors inherit many advantages, such as immunity to electromagnetic interference, compact size, high resolution, safety in hazardous environments, high sensitivity, low noise, high durability against seawater corrosion, the possibility of processing the signal at a long distance from the sensor with little degradation, etc. 

An optical fiber sensor for salinity measurement was proposed by Zhao et al. [9]. In the measurement, the deviation of the refracted beam caused by the change in the liquid refractive index was used as an indicator for the change in liquid salinity (PSD). An advanced fabrication technique, such as focused ion beam (FIB) milling, has been employed for manufacturing a three-wave Fabry-Pérot interferometer (FPI) in a single-mode fiber (SMF). The sensitivity matrix method was used for the simultaneous measurement of temperature and salinity of seawater [10]. Fiber Bragg grating (FBG) biosensors and chemical sensors [11,12,13,14] are important types of fiber sensors that have received much attention in biochemistry applications. In general, FBGs are functionalized with an RI-sensing ability through the removal of parts or the entire cladding of the fiber by means of post-treatment techniques, such as chemical etching and mechanical polishing, to enhance the evanescent field interaction with the analyte. Liang et al. reported a high-sensitivity RI sensor based on a 1.5 µm diameter etched eroded fiber FPI [15] that can detect an index variation as small as 1.4 × 10^−5^. Long-period grating coupling the core modes into the cladding modes results in the increased intensity of the evanescent field in the fiber cladding. Therefore, it can sense the ambient RI without etching the cladding or a complex structure design. Microfiber is another type of carrier frequently used to design optical sensors due to its very large exposed evanescent field. Many different structures with enhanced sensitivities have been developed [16]. Tapered plastic fiber optic sensors based on the intensity modulation technique have been implemented for salinity and cement paste curing temperature measurement [17]. Different sensor’s head designs, such as U-shaped and spiral-shaped, have been investigated and compared. U-shaped sensors have proven to be more efficient in terms of simplicity, sensitivity, and reversibility [18]. 

In this paper, a simple and low-cost salinity sensor based on a bundled plastic optical fiber (POF) and a concave mirror is demonstrated. The bundled POF comprises of a POF which serves as a transmitting fiber surrounded by ten smaller POFs, which serve as receiving fibers. A differential detection method is used to eliminate the fluctuation of the light source intensity and the photodetector’s (PD) transfer function. A compact multiplexing system with four integrated sensors is proposed. The multiplexing system comprises of a light-emitting diode (LED) as the light source and the high-speed photologic photodetectors. A He-Ne laser and silicon detector were used in the work of [19]. The cost of the proposed sensor system is reduced by approximately 70% compared with the early work reported in [19]. Experimental results indicate that the salinity detection sensitivity of the proposed sensor is 1279 times higher than the previously reported method in [19].

## 2. Analytical Results and Discussions

**Figure 1:**
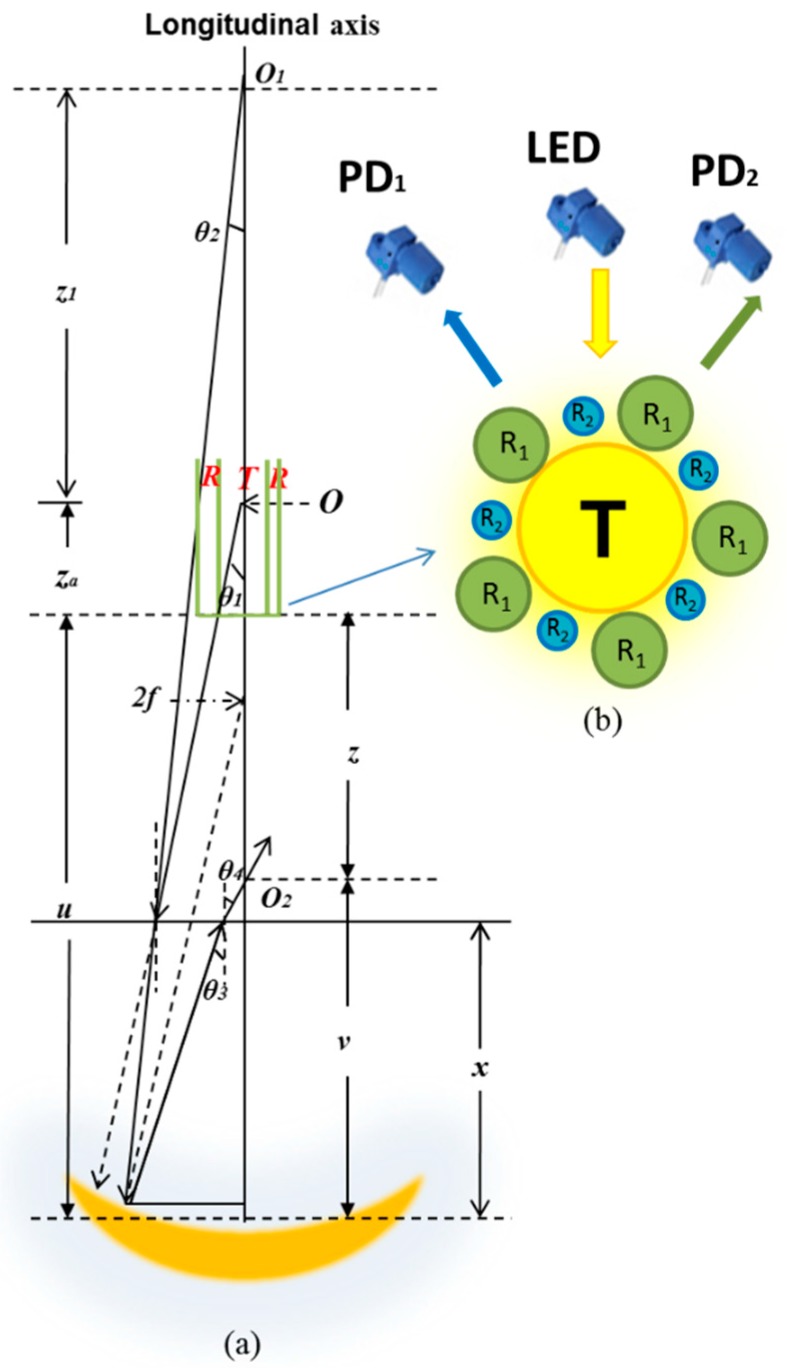
(**a**) Diagram of ray trace for proposed sensor; (**b**) The configuration of the sensor probe.

The geometrical configuration of the proposed sensor is shown in Figure 1a. The longitudinal axis of the transmitting fiber core is coaxial with the normal axis of the concave mirror. The original laser source emitting from point *O* is situated inside the transmitting fiber at a distance of *z_a_* from the fiber end with an emitting angle *θ*_1_. The emitted light incident on the liquid surface is refracted by an angle *θ*_2_. The liquid height is *x*. Light reflected by the air-liquid interface is very weak and its contribution to the received power is not significant. Based on the refracted ray geometry in the liquid, the virtual point source *O*_1_ is located at a point *z*_1_ along the longitudinal axis above the original laser source *O* in the air. The refracted light is then reflected by the concave mirror immersed in the liquid. The light converges at a point *O*_2_ and becomes the second virtual emitting point source for the receiving fiber. The location of the new virtual emitting point source *O*_2_ is influenced by the RI of liquid and the height of liquid *x*. The new virtual point source *O*_2_ emits at an angle *θ*_4_ with the longitudinal axis in the opposite direction.

Figure 1b illustrates the configuration of the sensor probe. It is a bundled POF that comprises of a transmitting fiber (T) and 10 receiving fibers. The light from the LED is guided by the transmitting fibers to the concave mirror, whereas the receiving fibers are used to collect the reflected light. Since they are made of the same material, all of the fibers are assumed to have the same transmission loss *K.* The receiving fibers are divided into two groups in which five of them (R_1_) are connected to PD_1_, whereas the other five (R_2_) are connected to PD_2_. As such, the power detected by PD_1_ and PD_2_ can be given by $P_{r(i)}=5P_{re(i)}$, where *P_r_*_1_ is the light power detected by PD_1_, *P_r_*_2_ is the light power detected by PD_2_, and *P_re_* is the light power collected by each receiving fiber. *P_re_* can be expressed as a function of liquid RI *n* and the height of liquid level *x*,

(1) $$P_{re}=\frac{2z^{2}P_{E}\left(\exp(-\frac{2r^{2}}{\theta_{4}^{2}{\left(u-v_{1}\right)}^{2}})-\exp(-\frac{D^{2}}{2\theta_{1}^{2}{\left(z_{a}+u\right)}^{2}}-\frac{2r^{2}}{\theta_{4}^{2}{\left(u-v_{1}\right)}^{2}})\right)}{\theta_{4}^{2}{\left(u-v_{1}\right)}^{2}}$$

where *r* and *z* are the radius and longitudinal coordinates along the receiving fiber, respectively; *D* is the diameter of the concave mirror; and the light power emitted from the transmitting fiber is *P_E_*. The expressions for *v*_1_ and *θ*_4_ are provided in [19]. It is worth noting that the receiving fibers, *R*_1_, have a larger core area diameter than that of *R*_2_. Both fiber groups will produce different characteristic curves. The intention of having two groups of receiving fibers with different core diameters will become clearer in the latter part of this section.

From the Beer-Lambert Law, we know that the light power in saline has an absorbance for *N* attenuating species in the solution, $A=\int_{0}^{l}\sum_{i=1}^{N}\varepsilon_{i}c_{i}(z)dz$, where *ε_i_* is the attenuation coefficient of the attenuating species *i* in the solution, *l* is the path length of the beam of light through the solution, and *c_i_* is the concentration of the attenuating species *i* in the solution. It is assumed that *PD*_1_ and *PD*_2_ have the same transfer function *F*, which converts the detected light powers of *P_r_*_1_ and *P_r_*_2_ to voltages *V*_1_ and *V*_2_, respectively. The output voltages of *PD*_1_ and *PD*_2_ can be expressed as:

(2) $$V_{1}=K_{1}F_{1}A_{1}P_{r1}$$

(3) $$V_{2}=K_{2}F_{2}A_{2}P_{r2}$$

If both receiving fibers are identical, then K_1_ = K_2_, F_1_ = F_2_. If the NaCl solution is uniform, it will induce the light absorbance inside the solution to only vary with the liquid level *x*. Hence, the light emitted from the second virtual emitting point source has a uniform absorbance, which means the absorbance *A*_1_ for detected *V*_1_ and *A*_2_ for detected *V*_2_ are identical. From the outputs of PD_1_ and PD_2_, a new characteristic output curve, *V* can be produced:

(4) $$V=\frac{V_{1}-V_{2}}{V_{1}+V_{2}}=\frac{P_{r2}-P_{r1}}{P_{r1}+P_{r2}}$$

For an intensity modulation-based fiber sensor, the sensor performance is heavily affected by the light source power intensity. However, the sensor’s output *V* in Equation (4) indicates that the sensor is immune to the variation of *K*, *F*, *P_E_*, and liquid absorption. According to Equation (4), we can see that the sensor output depends on the variables RI *n*, liquid level *x*, and sensor probe position *u*. For a fixed liquid level *x*, a small change of RI *n* or position *u* excites the variation of the sensor’s responses.

**Figure 2:**
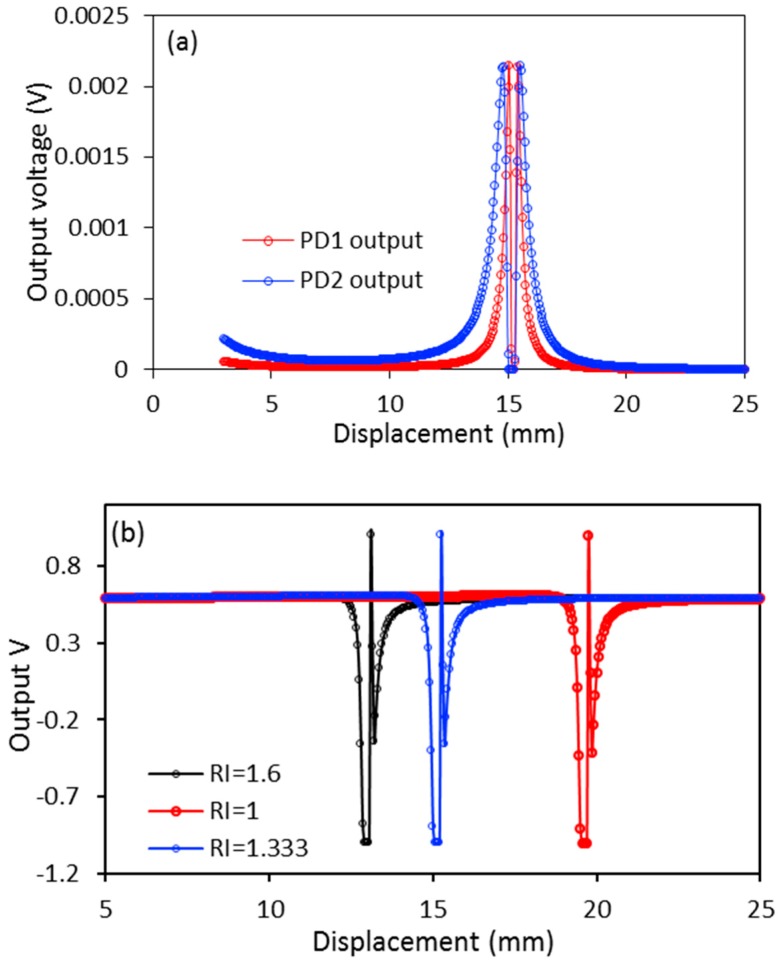
Analytical results of the proposed sensor: (**a**) sensor outputs of PD_1_ and PD_2_ for a liquid with RI = 1.333; (**b**) *V* (calculated using Equation (4)) for three RIs: 1, 1.333, and 1.6. Some of the important parameter settings used in the simulation are as follows: the wavelength of the laser source = 650 nm; numerical aperture NA = 0.35; fiber radii of T, R_1_, and R_2_ are 0.25 mm, 0.1 mm, and 0.05 mm, respectively. The focal lengths of concave mirrors are 10 mm and their diameters are 24 mm.

The analytical results of the proposed sensor are presented in Figure 2, of which, Figure 2a shows the sensor outputs of two PDs for RI = 1.333 and *u* is in the range of 3–25 mm. The near displacement region (0–3.0 mm) is referred to as the blind region for RI sensing because the same power variations are observed in the output responses for different RI. The difference can only be observed when the displacement goes beyond 3.0 mm. One dip located in between the two peaks is observed in each output curve. The dip is located at the position in which the displacement is twice the focal length of the concave mirror in the air medium (RI = 1). However, the dip position is closer to the mirror when the air medium is replaced with a liquid with a higher RI. At these points, the virtual point source *O*_2_ is located at the end facet of the transmitting fiber in which the incident light cone becomes a point at the center of the transmitting fiber core. This result is zero, or minimum reflective light, collected by the receiving fiber. 

From Equation (1), it can be seen that the light power detected by the PDs are related to the radius of the receiving fiber *r*. Two groups of receiving fibers with different core radii will induces the PDs to have slightly different output curves, which are shown in Figure 2a. The two output curves in Figure 2a indicate that a differential detection method (Equation (4)) can be employed to improve the sensors’ sensitivity. Figure 2b shows the output of the sensor for three different RIs using the differential detection method. Each curve in this figure has one dip and one peak, in which the dip is close to the zero point when the RI is increasing. The results in this figure also show that the proposed sensor has a very broad RI detection range. From the curves in Figure 2a, it can be seen that a slight change of displacement at ~15 mm will excite the PDs’ output to vary slowly. However, the curve (RI = 1.333) in Figure 2b shows that the slight change of *u* at ~15 mm will induce the output *V* to vary sharply. As such, it can be concluded that the proposed sensor’s sensitivity is improved significantly by employing two groups of receiving fibers with different core radii, combined with the differential detection method.

## 3. Experimental and Results Discussion

**Figure 3:**
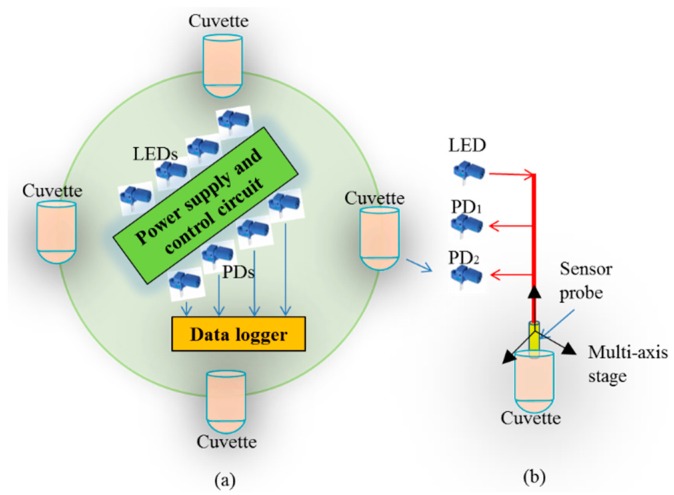
Experimental setup (**a**) multiplexing system for simultaneous detection of the salinity in four cuvettes; and (**b**) one sensor structure which is integrated in the multiplexing system.

The experimental setup of the proposed fiber sensor is shown in Figure 3a for simultaneous detection of the salinity in four cuvettes. In this system, four sensor probes are multiplexed into one system which integrates four LEDs and eight PDs to emit and detect the light, respectively, as shown in Figure 3a. Four concave mirrors with the same focal length of 10 mm and diameter of 24 mm are located at the bottom of the four cuvettes, respectively. Four sensor probes, as shown in Figure 1b, are formed with the POF with the same parameters, with that the radius of the transmitting fibers is 0.25 mm and the radii of the receiving fibers are 0.1 mm and 0.05 mm. The POFs have core radii of 0.09 mm and 0.045 mm, and refractive indices of 1.492 and 1.450 for the core and cladding, respectively. A circuit is designed to provide a stable current to drive the LEDs (red light, centered at 660 nm). The LEDs used in this setup show an excellent stability in the output light intensity. Figure 3b illustrates one of the sensor structures for salinity detection in a cuvette, in which the light is delivered through the sensor probe and collected by two high-speed photologic PDs. The PD converts the light power into a voltage signal. The circuit also equips a simple function to process the output voltages of the PDs to achieve the equation *V* = (*V*_1_ − *V*_2_)/(*V*_1_ + *V*_2_). Of which, *V*_1_ and *V*_2_ are the output voltages of PD_1_ and PD_2_, respectively. A TDS-530 data logger (Tokyo Sokki Kenkyuio Co. Ltd., Tokyo, Japan) is used to collect the processed output voltage signal *V*. The TDS-530 data acquisition system is set at a 640 mV detection range. The data logger is also set for the acquisition of one point each second. Four sensor probes are fastened on a multi-axial stage that provides a precise displacement control. The stage has a displacement resolution of 0.1 µm. The control of displacement in the y-axis direction is used to move the sensor probes in the vertical direction while the other two axes ensure accurate alignment between the longitudinal axis of the fiber probes and the normal axis of the center of the concave mirror. The experiment was conducted in a laboratory where the room temperature was 25 °C and the experimental setup includes four cuvettes, the multi-axial stage, and four sensor probes are placed on an optical table, a vibration-free platform for the experiment. To accurately control the liquid surface flatness, a wait time of 5 min is imposed after each movement of the liquid level or sensor probes to reduce the surface corrugations. Before the experiment, both NaCl salt and distilled water are stored at room temperature (25 °C) for two days to ensure the accuracy of experimental results. The solutions with concentrations of 1 wt % to 5 wt % with 1 wt % intervals were prepared to test the proposed sensor. The system drift is studied by employing the measurement system to detect the received light intensity. The room temperature is also monitored by using a thermocouple. The test is performed for 48 h, and a maximum drift of ~2% and 2.0 °C is found for the light intensity and room temperature, respectively. This indicates that the experimental work occurs under well-controlled ambient conditions.

**Figure 4:**
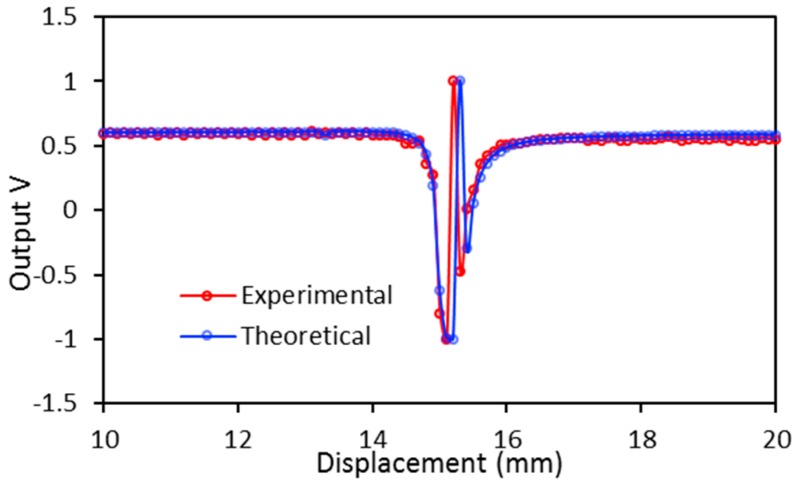
Theoretical and experimental results when the water RI = 1.333 and *x* = 3 mm for moving sensor probe *u*.

From Equation (4), it is worth noting that the liquid level *x* also affects the sensor response. However, in the practical application the liquid forms a convex surface at *x* > 3 mm when it is poured on the concave mirror. The liquid convex surface reflects and diverges the incident light from the emitting fiber. This results in a reduction in the light power collected by the receiving fiber. A minimum liquid height of 3 mm is suggested to avoid the occurrence of the convex liquid surface. The theoretical and experimental curves in Figure 4 are in good agreement with each other, except for the positions of the dip and the peak. It is shown that the dip and peak of the experimental curve is closer to the zero point than that of the theoretical case. This can be attributed to the concave liquid surface, while a flat surface is assumed in the theoretical approximation. The concave liquid surface yields a lower position of the virtual point source *O_2_* than that of a flat surface, which contributes the discrepancy between the theory and experiment. It is worth noting the liquid surface depends on the liquid level, *x*. However, its influence on the sensor output is minimal and it can be disregarded.

**Figure 5:**
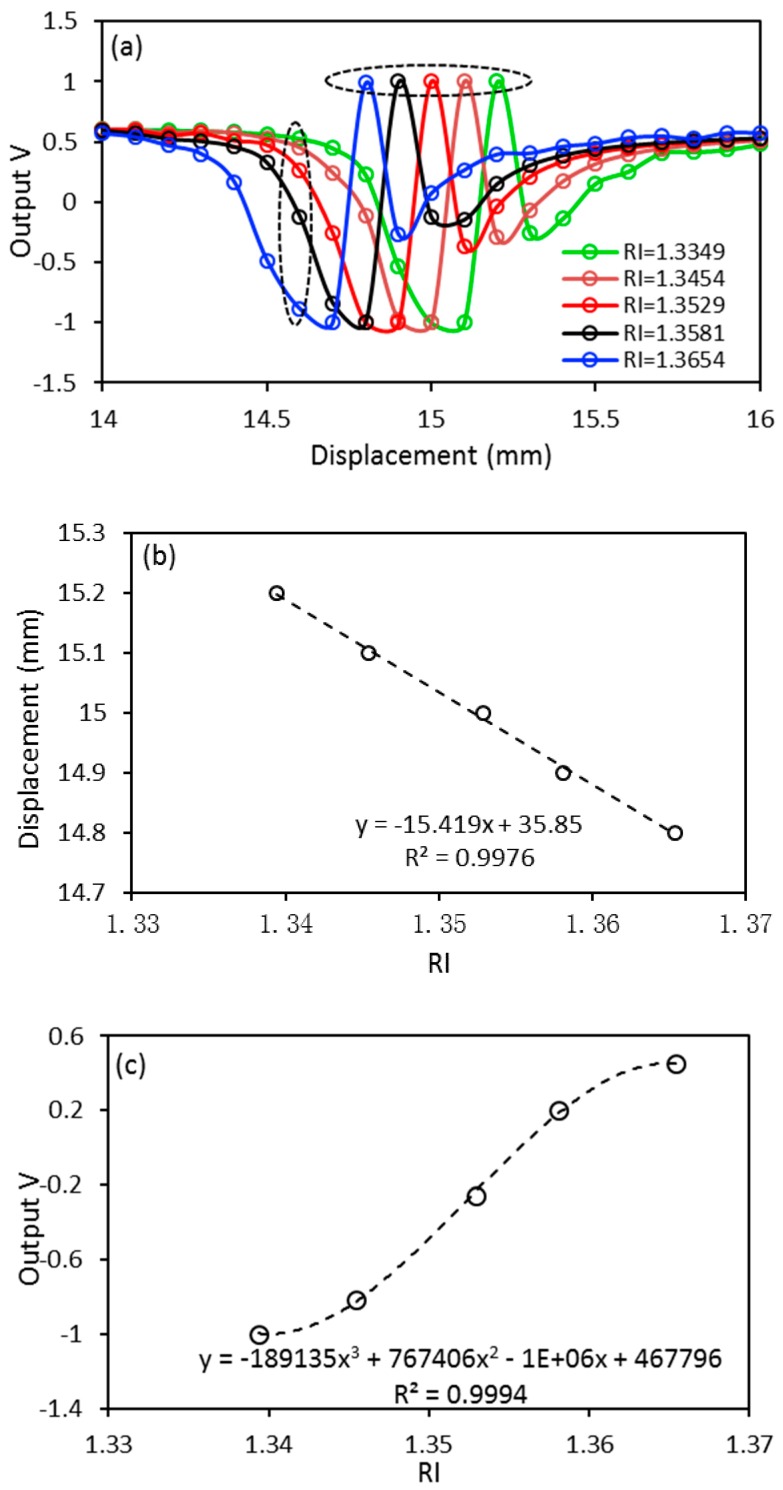
(**a**) Experimental results; and the sensitivities of the proposed sensor for (**b**) method 1 and (**c**) method 2.

The saline solution is slowly filled into the cuvette to make sure the level is as close to 3 mm as possible. The sensor probes are moved at a step size of 0.1 mm, between 3 mm and 25 mm, twice. After that the collected data are averaged. The measured RIs of the saline solutions by a prism coupler at 632.8 nm are 1.3394, 1.3454, 1.3529, 1.3581, and 1.3654, which increase with the higher salinity concentration. The calibrated experimental results are only plotted in the displacement of 14 mm and 16 mm, which includes five peaks and five dips, as shown in Figure 5a. The results in Figure 5a clearly show that the peak of the curve is moving closer to the zero point with the increasing RI, as enclosed in the horizontal dotted ellipse. This can be attributed to the lower virtual point source *O*_2_ produced from the solution with a higher RI. Furthermore, the readings at the displacement of 14.7 mm (refer to the vertical dotted ellipse in Figure 5a) also varies with the increase of the RI. As such, the points in these two regions can be used as the RI indicators which provide two RI detection approaches for the proposed sensor: (Method 1) a fixed liquid level *x* with varying sensor probe position, *u*, as indicated by the horizontal dotted ellipse; and (Method 2) a fixed sensor probe position, *u*, but varying the liquid level *x*, as indicated by the vertical dotted ellipse. Accordingly, the sensitivity of the proposed sensor is shown in Figure 5b,c for Methods 1 and 2, respectively. A linear curve fitting can be applied to the data points in Figure 5b for Method 1, which show that the proposed sensor has a sensitivity of 15.419 mm/RIU by using this method. From Figure 5c, it also can be found that the proposed sensor exhibits a maximum sensitivity of 14,847.486/RIU at RI = 1.3525 by using Method 2. The experimental setup in Figure 3a shows that the proposed sensor has an ability for the measurement of four samples simultaneously. The experiment was also performed with saline solutions with the same concentration to ensure the consistency of the test. The output data from the four sensors have a standard deviation of *σ* = 0.02.

To ensure the repeatability and accuracy of the measurements, re-calibration is performed prior the experiment. The sensor probe is reset to the same position in which distance *u* is the same in every measurement. This step can be treated as the zero setting. Then, the solution can be slowly poured into the cuvettes to increase the liquid level in steps from 0 mm to 3 mm during the re-calibration work. After each test, the sensor probe and the used cuvettes are cleaned with DI water and dried before proceeding to the next test. 

## 4. Conclusions

A high-sensitivity, low-cost optical fiber sensor is studied theoretically and demonstrated experimentally for salinity detection. Sensitivities of 15.419 mm/RIU and 14,847.486/RIU at RI = 1.3525 are, respectively, obtained for different detection methods with respect to the change of NaCl concentration. The experimental results also indicate that the proposed sensor is capable of simultaneous RI measurements of four samples. However, more improvement is required for the proposed sensor to meet the industrial expectation. Among the efforts include replacing the multi-axis stage with an automated translation stage to achieve automatic measurement, making the sensor probe and light source movable to meet the requirement of RI measurement at different wavelengths, and a re-design the sensor probe to achieve simultaneous measurement of temperature and RI. The influence of ambient temperature and mechanical perturbation on the performance of the sensor in the field outside of the laboratory will be investigated in the future.

## References

[1] Yang H., Wang S., Wang X., Wang J., Liao Y. (2014). Temperature Sensing in Seawater Based on Microfiber Knot Resonator. Sensors.

[2] Emmanuel A.O., Oladipo F.A., Olabode O. (2012). Investigation of salinity effect on compressive strength of reinforced concrete. J. Sustain. Dev..

[3] Le Menn M., de la Tocnaye J.D.B., Grosso P., Delauney L., Podeur C., Brault P., Guillerme O. (2011). Advances in measuring ocean salinity with an optical sensor. Meas. Sci. Tech..

[4] Yang H.Z., Qiao X.G., Ali M.M., Islam M.R., Lim K.S. (2014). Optimized tapered optical fiber for ethanol (C_2_H_5_OH) concentration sensing. J. Light. Technol..

[5] Luo D., Zainah I., Zubaidah I. (2013). Use of tapered optical fiber sensors in study of the hydration process of cement paste. IEEE Sens..

[6] Luo D., Ibrahim Z., Ma J., Ismail Z., Iseley D.T. (2016). Tapered Polymer Fiber Sensors for Reinforced Concrete Beam Vibration Detection. Sensors.

[7] Guzmánsepúlveda J.R., Guzmáncabrera R., Torrescisneros M., Sánchezmondragón J.J., Mayarrioja D.A. (2013). A highly sensitive fiber optic sensor based on two-core fiber for refractive index measurement. Sensors.

[8] Yoo W.J., Sim H.I., Shin S.H., Jang K.W., Cho S., Moon J.H., Lee B. (2014). A fiber-optic sensor using an aqueous solution of sodium chloride to measure temperature and water level simultaneously. Sensors.

[9] Yong Z., Bo Z., Yan B.L. (2003). Experimental research and analysis of salinity measurement based on optical techniques. Sens. Actuators B.

[10] Nguyen L.V., Vasiliev M., Alameh K. (2011). Three-wave fiber Fabry–Pérot interferometer for simultaneous measurement of temperature and water salinity of seawater. Photon. Technol. Lett..

[11] Wang G., Shum P.P., Ho H.P., Yu X., Hu D., Cui Y. (2011). Modeling and analysis of localized biosensing and index sensing by introducing effective phase shift in microfiber Bragg grating. Opt. Express.

[12] Schuster T., Herschel R., Neumann N., Schäffer C.G. (2012). Miniaturized long-period fiber grating assisted surface plasmon resonance sensor. J. Light. Technol..

[13] Luo D., Zainah I., Zubaidah I. (2013). Determination of early age setting time for cement pastes using a fiber Bragg grating sensor. Measurement.

[14] Yang H., Qiao X., Islam M., Lim K., Harith A. (2014). Simultaneous measurement of aliphatic alcohol concentration and temperature based on etched taper FBG. Sens. Actuators B.

[15] Liang W., Huang Y., Xu Y., Reginald K.L., Yariv A. (2005). Highly sensitive fiber bragg grating refractive index sensors. Appl. Phys. Lett..

[16] Yang H.Z., Qiao X.G., Luo D., Lim K.S., Chong W., Harun S.W. (2014). A Review of Recent Developed and Applications of Plastic Fiber Optic Displacement Sensors. Measurement.

[17] Luo D., Zainah I., Xu B., Zubaidah I. (2013). Optimization of the Geometries of Biconical Tapered Fibre Sensors for Early-age Curing Temperature Monitoring of Concrete Specimens. Comput. Aided Civ. Infrastruct. Eng..

[18] Wang J., Chen B. (2012). Experimental research of optical fiber sensor for salinity measurement. Sens. Actuators A.

[19] Yang H.Z., Qiao X.G., Lim K.S., Harun S.W., Chong W.Y., Islam M.R., Ahmad H. (2014). Optical fiber sensing of salinity and liquid level. IEEE Photonics Technol. Lett..

